# Atmospheric H_2_S and SO_2_ as sulfur source for *Brassica juncea* and *Brassica rapa*: impact on the glucosinolate composition

**DOI:** 10.3389/fpls.2015.00924

**Published:** 2015-10-28

**Authors:** Tahereh Aghajanzadeh, Stanislav Kopriva, Malcolm J. Hawkesford, Anna Koprivova, Luit J. De Kok

**Affiliations:** ^1^Laboratory of Plant Physiology, Groningen Institute for Evolutionary Life Sciences, University of GroningenGroningen, Netherlands; ^2^Botanical Institute and Cluster of Excellence on Plant Sciences, Cologne Biocenter, University of CologneCologne, Germany; ^3^Plant Biology and Crop Science Department, Rothamsted ResearchHarpenden, UK

**Keywords:** atmospheric sulfur nutrition, *Brassica juncea*, *Brassica rapa*, glucosinolates, hydrogen sulfide, sulfate nutrition, sulfate deprivation, sulfur dioxide

## Abstract

The impact of sulfate deprivation and atmospheric H_2_S and SO_2_ nutrition on the content and composition of glucosinolates was studied in *Brassica juncea* and *B. rapa*. Both species contained a number of aliphatic, aromatic and indolic glucosinolates. The total glucosinolate content was more than 5.5-fold higher in *B. juncea* than in *B. rapa*, which could solely be attributed to the presence of high levels of sinigrin, which was absent in the latter species. Sulfate deprivation resulted in a strong decrease in the content and an altered composition of the glucosinolates of both species. Despite the differences in patterns in foliarly uptake and metabolism, their exposure hardly affected the glucosinolate composition of the shoot, both at sulfate-sufficient and sulfate-deprived conditions. This indicated that the glucosinolate composition in the shoot was hardly affected by differences in sulfur source (viz., sulfate, sulfite and sulfide). Upon sulfate deprivation, where foliarly absorbed H_2_S and SO_2_ were the sole sulfur source for growth, the glucosinolate composition of roots differed from sulfate-sufficient *B. juncea* and *B. rapa*, notably the fraction of the indolic glucosinolates was lower than that observed in sulfur-sufficient roots.

## Introduction

Brassicaceae are nutritionally important crops containing relatively high levels of sulfur-containing secondary metabolites, viz., glucosinolates, which are not only responsible for the flavor of these species but also may be of great significance as phytopharmaceuticals considering their potential anti-carcinogenic properties (Fahey et al., 2001; Wittstock and Halkier, 2002; Jahangir et al., 2009). The glucosinolate content varies strongly between *Brassica* species and in seedlings may account for 10–30 % of the organic sulfur fraction (Castro et al., 2004; Aghajanzadeh et al., 2014). Glucosinolates contain 2-3 S groups per molecule and have a common core structure of a β-D-thioglucose group linked to a sulfonated aldoxime moiety and a variable side chain derived from an amino acid (Wittstock and Halkier, 2002). Cysteine, the end product of the sulfate reduction pathway in the chloroplasts (and plastids in the root), functions as the reduced sulfur donor for the synthesis of glucosinolates. Moreover, 3′-phosphoadenosine 5′-phosphosulfate (PAPS), which is synthetized from adenosine 5′-phosphosulfate (APS), the first intermediate in the sulfate reduction pathway by APS kinase, is essential for the synthesis of the sulfated moiety of glucosinolates (Schnug, 1990, 1993; Halkier and Gershenzon, 2006; Falk et al., 2007; Kopriva et al., 2012). *Brassica* species contain a wide variety of glucosinolates, which on the basis of amino acid precursors, side chain elongation and further modification are classified in aliphatic, indolic, and aromatic glucosinolates (Wittstock and Halkier, 2002; Halkier and Gershenzon, 2006).

The content and composition of the glucosinolate pool in Brassicaceae depends on the developmental stage of the plant (Falk et al., 2007; Agneta et al., 2014). For instance, the highest glucosinolate contents were found in seeds, siliques and young leaves, while intermediate contents were found in leaves, stems and roots (Petersen et al., 2002; Brown et al., 2003). The composition of the glucosinolate pools in plant tissue appears be the consequence of *in situ* synthesis and/or their redistribution via long-distance transport (Matsuda et al., 2010; Andersen et al., 2013). Glucosinolates may be transported in plants via the xylem (Sattelmacher, 2001) and the phloem (Lucas et al., 2013) and recently two glucosinolate-specific transporters, GTR1 and GTR2, have been identified, which are involved in the long-distance inter-organ transport (Andersen et al., 2013).

It has been presumed that glucosinolates would have significance in the storage of sulfur and that at sulfur-deprived conditions these compounds would be degraded by myrosinase in order to enable the re-distribution of sulfur in plants (Schnug, 1990; Hirai et al., 2004, 2005; Bloem et al., 2007; Falk et al., 2007). However, SO_2_ and H_2_S exposure did not affect the glucosinolate content of *Brassica juncea* and *B. rapa*, which are characterized by a relatively high and low glucosinolate content, respectively, showing that these sulfur-containing secondary metabolites did not form a sink for excessive atmospheric sulfur supply (Aghajanzadeh et al., 2014). Whilst sulfate deprivation resulted in a decrease in the glucosinolate content of *Brassica* seedlings, the proportion in the organic sulfur fraction was higher than that of sulfate-sufficient plants, even upon SO_2_ and H_2_S exposure, indicating that in *Brassica* seedlings glucosinolates had hardly any significance in the re-distribution of sulfur upon sulfate deprivation (Aghajanzadeh et al., 2014).

In the current paper, the impact of the plants’ sulfur source for growth, viz., sulfate taken up by the root, and/or SO_2_ and H_2_S taken up by the shoot, on the content and composition of glucosinolates was studied in detail in shoots and roots of *B. juncea* and *B. rapa*.

## Materials and Methods

### Plant Material and H_2_S and SO_2_ Exposure

Seeds of *B. juncea* cv. Rugosa and *B. rapa* cv. Komatsuna (Van der Wal, Hoogeveen, The Netherlands) were germinated in vermiculite in a climate-controlled room. Day and night temperatures were 22 and 18°C (±1°C), respectively, relative humidity was 60–70%. The photoperiod was 14 h at a photon fluence rate of 300 ± 20 μmol m^-2^ s^-1^ (400–700 nm) at plant height, supplied by Philips GreenPower LED (deep red/white 120) production modules. 10 day-old seedlings were transferred to an aerated 25% Hoagland nutrient solution at 0.5 mM sulfate for 3 days and subsequently transferred to fresh Hoagland nutrient solution at 0 mM sulfate (-S, sulfate-deprived) or 0.5 mM sulfate (+S, sulfate-sufficient) in 13 l stainless steel containers (30 plants per container). Plants were exposed to 0.25 μl l^-1^ H_2_S or SO_2_ for 7 days in 150 l cylindrical stainless steel cabinets (0.6 m diameter) with a polymethyl methacrylate top. Sealing of the lid of the containers and plant sets prevented absorption of atmospheric H_2_S or SO_2_ by the solution. Day and night temperatures were 24 and 20°C (±2°C), respectively, and relative humidity was 40–50%. The photoperiod was 14 h at a photon fluence rate of 300 ± 20 μmol m^-2^ s^-1^ (400–700 nm) at plant height, supplied by Philips GreenPower LED (deep red/white 120) production modules. The temperature inside the cabinets was controlled by adjusting the cabinet wall temperature. See for further details Aghajanzadeh et al. (2014). Plants were harvested 3 h after the onset of the light period and the roots were rinsed in ice-cold demineralized water (for 3 s × 20 s). Roots were separated from the shoots, weighed, and for glucosinolate analyses, plant material was frozen immediately in liquid N_2_ and stored at -80°C.

### Glucosinolate Content and Composition

The glucosinolates contents were analyzed with reverse phase HPLC and UV detection method, as described by Burow et al. (2006). The glucosinolates were extracted at 70°C from 50 mg freeze-dried plant material in 70% methanol (v:v) for 45 min. The extract was centrifuged for 5 min at 13,000 *g*. After centrifugation, 1 ml of the supernatant was loaded on a DEAE Sephadex A-25 column. The column was washed with 2 ml × 0.5 ml water and 20 mM sodium acetate. After washing, the column was treated overnight with sulfatase to convert the glucosinolates to their desulfated derivatives. The desulfated glucosinolates were eluted off the column with 2 ml × 0.5 ml water and were separated by reverse-phase HPLC using an ODS2 column (Waters) and a gradient of acetonitrile in water (5–30% in 8 min, 30–50% in 7 min). The desulfo-glucosinolates were detected and quantified by UV absorption at 229 nm relative to sinigrin used either as internal standard (for *B. rapa*) or as standard curve (for *B. juncea*) using response factors. The individual desulfo-glucosinolates were identified based on retention times and known profiles.

### Statistical Analysis

Data from different experimental sets ware analyzed for statistical significance using an unpaired two-tailed Student’s *t*-test (*P* < 0.01).

## Results

### Impact of Sulfur Nutrition on Glucosinolate Content

**FIGURE 1:**
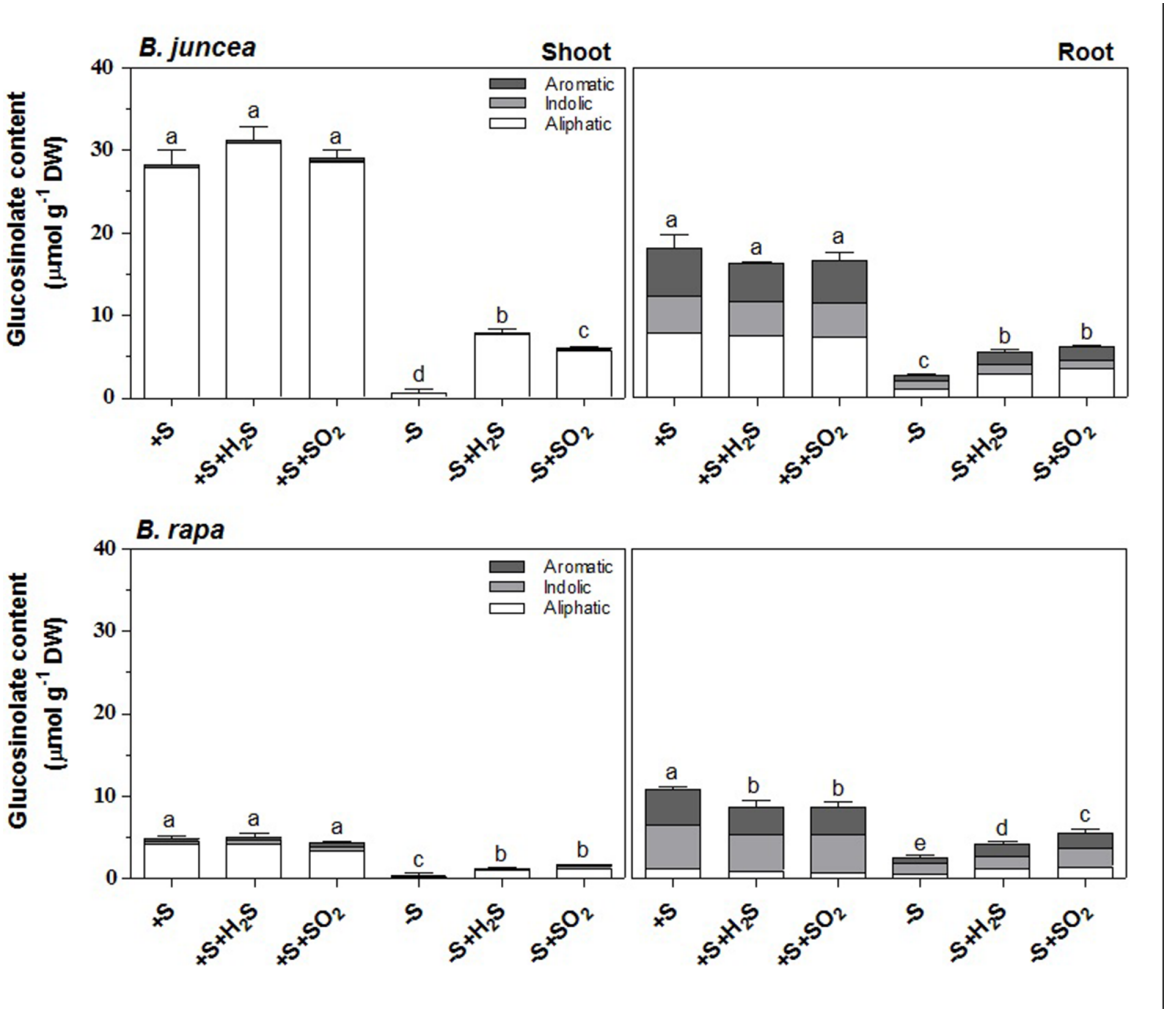
**Impact of H_2_S and SO_2_ and sulfate deprivation on the content and composition of glucosinolates in shoots and roots of *Brassica juncea* and *B. rapa*.** 10 day-old seedlings were grown on a 25 % Hoagland solution containing 0.5 mM sulfate for 3 days and subsequently transferred to fresh 25% Hoagland solution at 0 (-S) or 0.5 mM sulfate (+S) and exposed to 0.25 μl l^-1^ H_2_S or SO_2_ for 7 days. The content of the aliphatic (white bars; μmol g^-1^ DW), indolic glucosinolates (gray bars; μmol g^-1^ DW) and aromatic (dark gray bars; μmol g^-1^ DW) represent the mean of three measurements with nine plants in each (±SD). Different letters indicate significant differences between treatments (*P* < 0.01, Student’s *t*-test).

Similarly to previous observations (Aghajanzadeh et al., 2014), the glucosinolate content was substantially higher in shoots of *B. juncea* than *B. rapa* seedlings (5.5-fold), but it was not affected upon a 7-day exposure to 0.25 μl l^-1^ H_2_S or 0.25 μl l^-1^ SO_2_ (**Figure 1**). Sulfate-deprivation resulted in strongly decreased glucosinolate content, both in shoots and roots of *B. juncea* and *B. rapa* (Aghajanzadeh et al., 2014; **Figure 1**). The glucosinolate content of both shoot and root of sulfate-deprived plants was substantially enhanced upon H_2_S or SO_2_ exposure, although was still lower than that observed in sulfate-sufficient plants (Aghajanzadeh et al., 2014; **Figure 1**).

### Glucosinolate Composition

**FIGURE 2:**
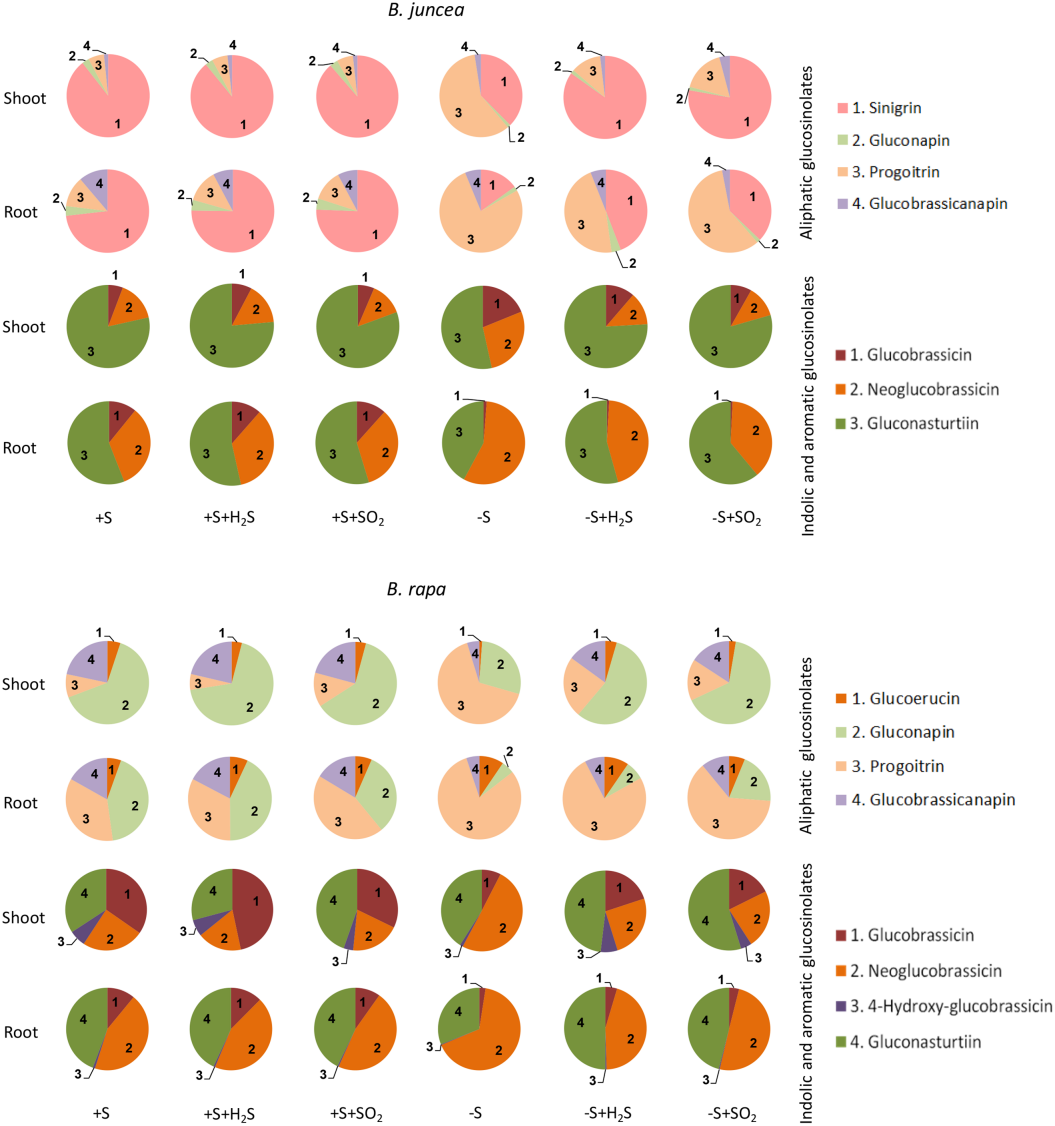
**Impact of H_2_S and SO_2_ and sulfate deprivation on the composition of glucosinolates in shoots and roots of *B. juncea* and *B. rapa*.** For experimental details, see legends of **Figure 1**. The pie graphs represent the composition of the glucosinolates (for details on their nomenclature see **Table 1**), the absolute data are presented in **Figures 3** and **4**.

Seven different glucosinolates could be identified in shoots and roots of *B. juncea;* three were short-chain (C3–C4) aliphatic glucosinolates (sinigrin, gluconapin and progoitrin), one long-chain (C5) aliphatic glucosinolate (glucobrassicanapin), two indolic glucosinolates (glucobrassicin, neoglucobrassicin) and the aromatic glucosinolate gluconasturtiin; **Table 1**, **Figure 2**). All of these glucosinolates, except sinigrin were also detected in the shoots and roots of *B. rapa* (**Table 1** and **Figure 2**). Roots and shoots of *B. rapa* also contained glucoerucin (an aliphatic glucosinolate) and 4-hydroxy-glucobrassicin (an indolic glucosinolate; **Figure 2**). The aliphatic glucosinolates were the predominant secondary sulfur compounds present in the shoots of both *B. juncea and B. rapa*, and they accounted for more than 98 and 84% of the total glucosinolates, respectively (**Figure 1**).

**Table 1:** Nomenclature of the individual glucosinolates identified in shoots and roots of *Brassica juncea* and *B. rapa.*

GSL type	Trivial name	Chemical name
Aliphatic	Sinigrin (3C)^∗^	2-Propenyl GSL
	Glucoerucin (4C)^∗^	4-Methylthiobutyl GSL
	Gluconapin (4C)^∗^	3-Butenyl GSL
	Progoitrin (4C)^∗^	2-Hydroxy-3-butenyl GSL
	Glucobrassicanapin (5C)^∗^	4-Pentenyl GSL
Indolic	Glucobrassicin	Indol-3-ylmethyl GSL
	Neoglucobrassicin	1-Methoxy-indol-3-ylmethyl GSL
	4-Hydroxyglucobrassicin	4-Hydroxy-indol-3-ylmethyl GSL
Aromatic	Gluconasturtiin	2-Phenylethyl GSL

*^∗^Carbon side chain. The individual glucosinolates are classified by trivial name and chemical name.*

**FIGURE 3:**
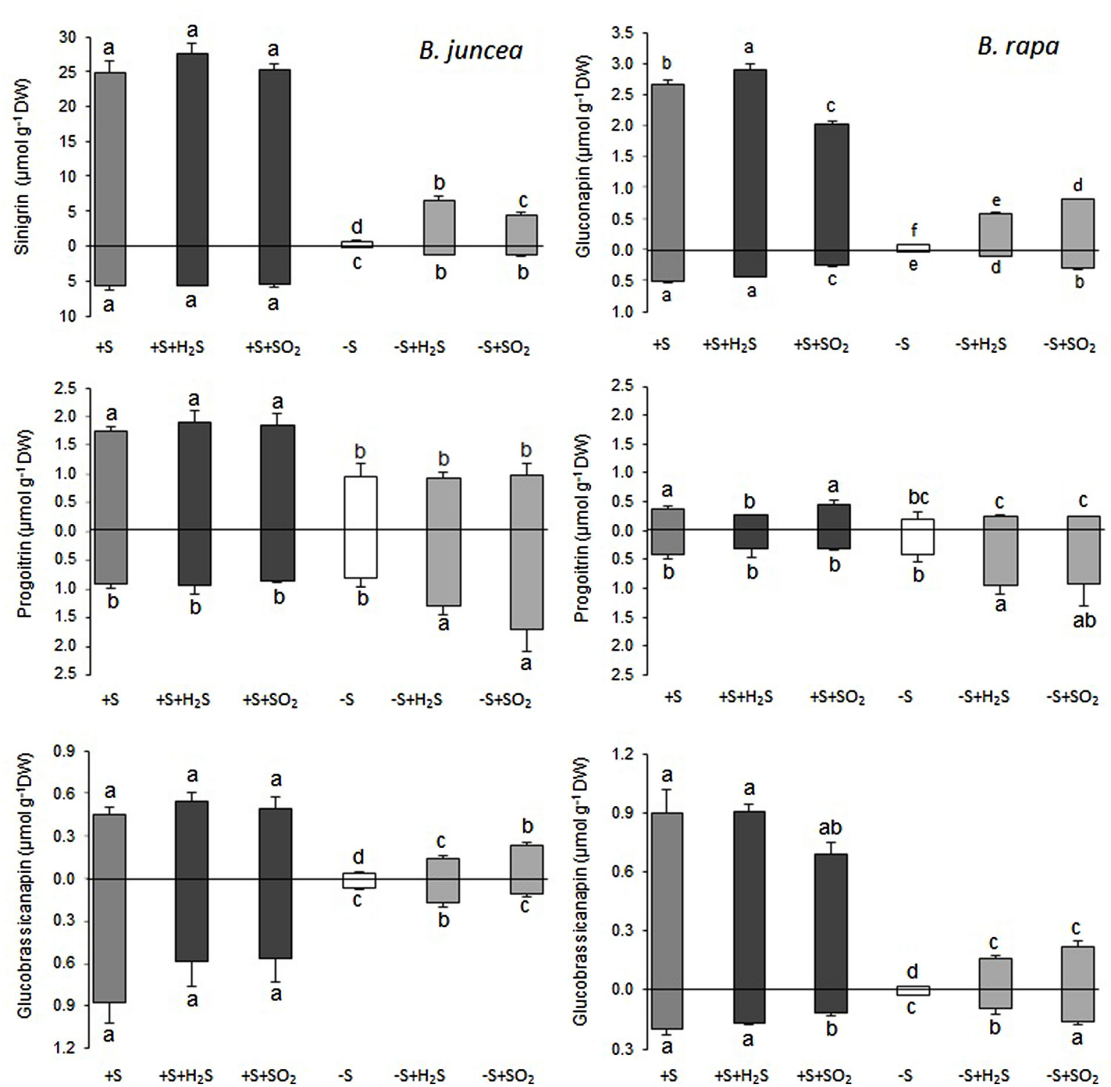
**Impact of H_2_S and SO_2_ and sulfate deprivation on the content of the aliphatic glucosinolates in shoots (above x axis) and roots (below x axis) of *B. juncea* and *B. rapa*.** For experimental details, see legends of **Figure 1**; for details on their nomenclature see **Table 1**. Data on glucosinolate content (μmol g^-1^ DW) represent the mean of three measurements with nine plants in each (±SD). Different letters indicate significant differences between treatments (*P* < 0.01, Student’s *t*-test).

**FIGURE 4:**
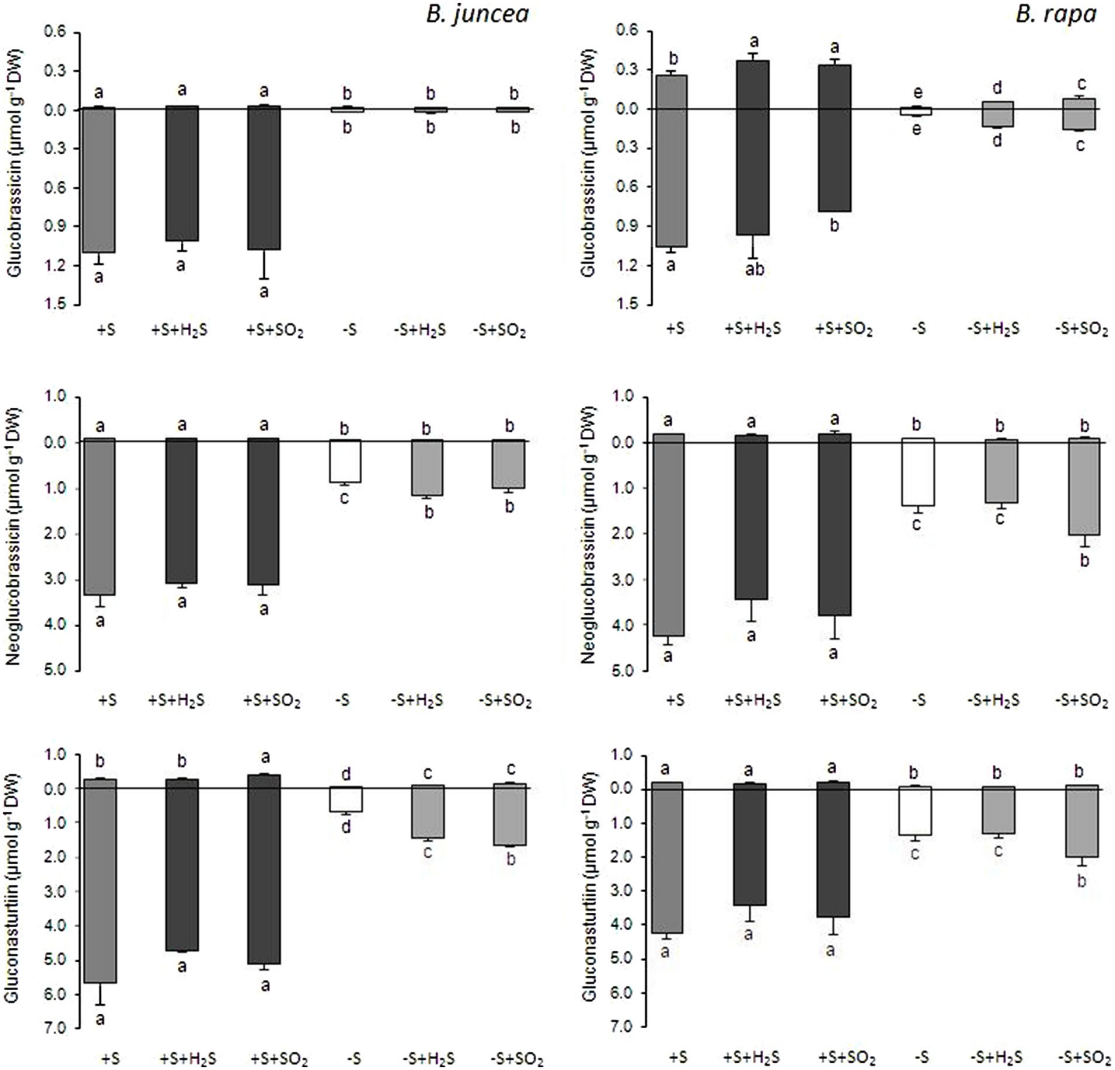
**Impact of H_2_S and SO_2_ and sulfate deprivation on the content of the indolic and aromatic glucosinolates in shoots (above x axis) and roots (below x axis) of *B. juncea* and *B. rapa*.** For experimental details, see legends of **Figure 1**; for details on their nomenclature see **Table 1**. Data on glucosinolate content (μmol g^-1^ DW) represent the mean of three measurements with nine plants in each (±SD). Different letters indicate significant differences between treatments (P < 0.01, Student’s *t*-test).

Sinigrin was the major aliphatic glucosinolate present in the roots and shoots of *B. juncea*. In the shoots it even accounted for more than 90% of the aliphatic and more that 80% of the total glucosinolates (**Figures 2** and **3**). The observed 5.5-fold higher glucosinolate content in the shoots of *B. juncea* was for the greater part attributed to the high sinigrin content, a compound which was not detected in the shoots and roots of *B. rapa* (**Figures 2** and **3**). Gluconapin was the major aliphatic glucosinolate present in roots and shoots of *B. rapa.* In the shoot its content accounted for 65% of the aliphatic glucosinolates and 57% of the total glucosinolates (**Figures 2** and **3**). The roots of both species, however, contained high contents of aromatic glucosinolates, which in accounted for 32 and 40% and indolic glucosinolates, which content accounted for 56 and 50% of the total glucosinolates in roots of *B. juncea and B. rapa*, respectively (**Figure 1**). Gluconasturtiin was the major glucosinolate in roots of *B. juncea* and *B. rapa* (**Figures 2** and **4**).

### Impact of Sulfur Nutrition on Glucosinolate Composition

H_2_S and SO_2_ exposure did not affect the total content, and only slightly affected the glucosinolate composition in the shoots and roots of sulfate-sufficient *B. rapa* seedlings (**Figures 1**–**4**). H_2_S exposure resulted in a slight increase in the gluconapin and glucobrassicin content and slight decrease in the progoitrin in the shoots of *B. rapa*, whereas it did not affect the aliphatic and indolic glucosinolate composition in the roots (**Figure 2**–**4**). SO_2_ exposure resulted in a slight increase in the glucobrassicin content and a decrease in the gluconapin and glucobrassicanapin content of the shoots of *B. rapa.*

A 7-day sulfate deprivation resulted in 96 and 85% decreases in total glucosinolate content in shoots and roots of *B. juncea* and 90 and 76% decreases in shoots and roots of *B. rapa* seedlings, respectively (**Figure 1**). Sulfate deprivation also strongly affected the glucosinolate composition and resulted in a 94, 74, and 82% decrease in the content of the aliphatic, indolic, and aromatic glucosinolates in the shoot and a 84, 85, and 88% decrease of these glucosinolates in the roots of *B. juncea*, respectively (**Figure 1**). In *B. rapa*, the content of aliphatic, indolic, and aromatic glucosinolates decreased by 93, 79, and 97% in the shoots, and by 55, 73, and 84% in the roots, respectively, upon sulfate deprivation (**Figure 1**). Evidently, the content of progoitrin and neoglucobrassicin in the shoots and roots of *B. juncea* and *B. rapa* were less affected by sulfate deprivation than that of the other glucosinolates (**Figures 3** and **4**).

Exposure of sulfate-deprived *B. juncea* seedlings to 0.25 μl l^-1^ H_2_S or SO_2_ partly alleviated the decrease in the glucosinolate content in both shoots and roots, but was always lower than that observed in sulfate-sufficient plants (**Figure 1**). Moreover, exposure of sulfate-deprived *B. rapa* seedlings to 0.25 μl l^-1^ H_2_S and SO_2_ partly alleviated the decrease in the glucosinolate content in the roots, whereas that of the shoots hardly changed (**Figure 1**). Again, sinigrin was the most abundant glucosinolate in the shoots of *B. juncea* and the overall glucosinolate composition in the shoot of this species was quite similar to that observed in sulfate-sufficient plants both in presence and absence of H_2_S and SO_2_ (**Figures 2** and **3**). Gluconapin was the most abundant glucosinolate present in the shoots of sulfate-deprived *B. rapa* upon H_2_S and SO_2_ exposure, and the overall glucosinolate composition was somewhat altered to that observed in sulfate-sufficient plants in presence and absence of H_2_S and SO_2_, due to the relatively higher content of this glucosinolate (**Figures 2** and **3**). In both sulfate-deprived H_2_S- and SO_2_-exposed *B. juncea* and *B. rapa* plants, the short-chain (C3) and (C4) aliphatic glucosinolates were predominant glucosinolates present in the shoots (**Figure 3**). Exposure of sulfate-deprived *B. juncea* to H_2_S and SO_2_ resulted in a substantial enhancement in the sinigrin content of the root, whereas the content of the other glucosinolates were less affected (**Figures 2** and **3**). As a consequence, the overall composition of the glucosinolates in H_2_S and SO_2_ exposed sulfate-deprived *B. juncea* roots was somewhat different than that observed in sulfate-sufficient plants (**Figure 2**). The composition of the glucosinolates of sulfate-deprived roots of *B. rapa* upon H_2_S and SO_2_ exposure was also different to that observed in sulfate-sufficient plants (**Figure 2**). The latter was mainly due to the overall higher proportion of progoitrin in the roots (**Figures 2** and **3**). H_2_S and SO_2_ exposure largely alleviated the decrease in short-chain (C4) aliphatic glucosinolates in roots of both *B. juncea* and *B. rapa* upon sulfate-deprivation.

## Discussion

Brassicaceae are able to utilize foliarly absorbed atmospheric H_2_S or SO_2_ as sulfur sources for growth which may replace sulfate taken up by the root as the sulfur source for growth (Stuiver and De Kok, 2001; Buchner et al., 2004; Yang et al., 2006; De Kok et al., 2007; Koralewska et al., 2008; Shahbaz et al., 2013; Aghajanzadeh et al., 2014). It was evident, that an atmospheric concentration of 0.25 μl l^-1^ of these sulfur gasses was sufficient to cover the sulfur requirements for growth. At an ample sulfate supply, the total sulfur contents of the plants were hardly affected, indicating a good coordination between uptake and assimilation of these sulfur gasses in the shoot and uptake of sulfate by the root and exposure of Brassicaceae to atmospheric sulfur resulted to a down-regulation of the sulfate uptake by the root and its reduction in the shoot (Westerman et al., 2000, 2001; Buchner et al., 2004; De Kok et al., 2007; Durenkamp et al., 2007; Koralewska et al., 2008; Shahbaz et al., 2013).

Despite the differences in the uptake of H_2_S and SO_2_ in the shoots (the uptake of H_2_S is determined by the rate of metabolism into cysteine, whereas that of SO_2_ by dissociation in the aqueous phase of the mesophyll), their impact at an atmospheric concentration of 0.25 μl l^-1^ on the total sulfur content of *B. juncea* and *B. rapa* seedlings was quite similar for both sulfate-sufficient and sulfate-deprived conditions (Aghajanzadeh et al., 2014). Neither H_2_S nor SO_2_ exposure affected the glucosinolate contents of shoots and roots of these species, demonstrating that these sulfur compounds did not form a sink for the storage of excessive sulfur, at least at a seedling stage (Aghajanzadeh et al., 2014). From the present study it was again evident that the shoots of *B. rapa* contained considerably lower levels of glucosinolates compared to *B. juncea*, although the differences in contents were mainly due to the high levels of sinigrin, which was absent in *B. rapa*. Moreover, the transcript level of APS kinase, a key enzyme in the synthesis of the sulfate moiety of the glucosinolates, was also substantially higher in shoots and roots of *B. juncea* (data not shown). However, H_2_S and SO_2_ exposure also hardly affected the composition of the glucosinolates.

Sulfate-deprivation of *B. juncea* and *B. rapa* for a week resulted in stunted growth and diminished sulfur and glucosinolate contents (Aghajanzadeh et al., 2014). The exposure of sulfate-deprived plants to 0.25 μl l^-1^ H_2_S or SO_2_ fully alleviated the decrease in biomass production, demonstrating that plants to utilize these atmospheric sulfur gasses as sole sulfur sources for growth. Again, despite the differences in patterns of uptake and metabolism between H_2_S and SO_2_, their impact on sulfur and glucosinolate contents in shoots and roots of *B. juncea* and *B. rapa* were quite similar. Their contents were lower than that observed in sulfur-sufficient plants in absence or presence of H_2_S and SO_2_ (Aghajanzadeh et al., 2014), although the proportion of the glucosinolates in the organic sulfur fraction was higher than that of sulfate-sufficient plants (Aghajanzadeh et al., 2014). From the current results it was evident that sulfate deprivation not only decreased the total glucosinolates contents but also strongly affected the composition. However, if sulfate-deprived plants were exposed to H_2_S and SO_2_, the composition of the glucosinolates in the shoots of both *B. juncea* and *B. rapa* hardly differed from that of sulfate-sufficient plants. Remarkably, the pattern of synthesis of the different aliphatic and indolic glucosinolates was hardly affected by the differences in oxidation state of the supplied sulfur sources in the shoots. Apparently, upon absorption and subsequent metabolism of H_2_S and SO_2_ in the shoots, not only sufficient cysteine, which functions as the reduced sulfur donor for the synthesis of glucosinolates, but also sufficient sulfate was formed/available for the synthesis of the sulfate moiety of glucosinolates via the ATP sulfurylase/APS kinase/sulfotransferase pathway. Evidently, foliarly absorbed SO_2_ may, after its reaction with water and dissociation in the mesophyll apoplast and symplast, be either non-enzymatically and/or enzymatically oxidized to sulfate or reduced in the chloroplast, and subsequently assimilated into cysteine (De Kok, 1990; De Kok and Tausz, 2001; De Kok et al., 2007). The direct metabolism of the foliarly absorbed H_2_S by *O-*acetyl(thiol)lyase, the rate limiting step for the uptake of these gas by the shoot, should provide ample cysteine as reduced sulfur donor for the synthesis of glucosinolates (De Kok, 1990; De Kok and Tausz, 2001; Stuiver and De Kok, 2001; De Kok et al., 2007). However, the source of sulfate moiety under these conditions needs further to be evaluated. The pattern of H_2_S oxidation in plant tissues is rather obscure; the presence of superoxide may catalyze the oxidation of sulfide, though it is still unclear to what extent elemental sulfur or sulfate is formed (De Kok et al., 1983). It has been observed that degradation in cysteine in cells may result in the formation of sulfate, although here the pathway also needs further to be investigated (Harrington and Smith, 1980).

The glucosinolate composition of sulfate-deprived roots after H_2_S and SO_2_ exposure was different from that observed in sulfate-sufficient *B. juncea* and *B. rapa.* In sulfate-deprived plants exposed to H_2_S or SO_2_, the roots fully depend on the sulfur supplied by the shoots. Roots of *B. juncea* and *B. rapa* express all enzymes of the sulfate reduction pathway and APS kinase (data not shown) which is essential for the synthesis of the sulfate moiety of the glucosinolates, indicating that the roots have the capacity to synthesize glucosinolates, despite the observation that they might also be transported in plant tissue (Andersen et al., 2013). It is widely accepted that in the majority of plant species glutathione is most important form of reduced sulfur transported from source (viz., shoot) to sink (root; Rennenberg et al., 1979; Herschbach et al., 2000). However, the pathway of degradation of glutathione, which, e.g., would be necessary for the synthesis of the sulfated moiety (via the APS/PAPS pathway) of glucosinolates in the roots, is still not well understood (Ohkama-Ohtsu et al., 2008).

## Conclusion

The glucosinolate composition in the shoot was hardly affected by differences in sulfur source (viz., sulfate, sulfite and sulfide) for growth, whereas that in the root was substantially altered. The latter indicated that the presence of sulfate in the root environment is essential for the synthesis of some of the glucosinolates.

## Conflict of Interest Statement

The authors declare that the research was conducted in the absence of any commercial or financial relationships that could be construed as a potential conflict of interest.
